# PD-L1 expression in testicular germ cell tumors undergoing spontaneous regression

**DOI:** 10.17305/bb.2024.10745

**Published:** 2024-12-01

**Authors:** Ivan Novak, Miroslav Tomić, Denis Mulabdić, Ivan Pezelj, Slaven Čiček, Igor Tomašković, Nino Sinčić, Božo Krušlin, Monika Ulamec

**Affiliations:** 1 School of Medicine, University of Zagreb, Zagreb, Croatia; 2 Department of Urology, University Hospital Centre Sisters of Charity, Zagreb, Croatia; 3 Emergency Medicine, General Hospital Dubrovnik, Dubrovnik, Croatia; 4 Department of Surgery, University Hospital Centre Sisters of Charity, Zagreb, Croatia; 5 Faculty of Medicine Osijek, Josip Juraj Strossmayer University of Osijek, Osijek, Croatia; 6 Department of Biology, School of Medicine, University of Zagreb, Zagreb, Croatia; 7 Scientific Centre of Excellence for Reproductive and Regenerative Medicine, School of Medicine, University of Zagreb, Zagreb, Croatia; 8 Clinical Department of Pathology and Cytology Ljudevit Jurak, University Hospital Centre Sisters of Charity, Zagreb, Croatia; 9 Department of Pathology, School of Medicine, University of Zagreb, Zagreb, Croatia

**Keywords:** Germ cell tumor, testis, spontaneous tumor regression, programmed death-ligand 1 (PD-L1), lymphocyte, biomarker, immunotherapy

## Abstract

Spontaneous regression of testicular germ cell tumors is a well-known phenomenon; however, the precise mechanisms of spontaneous regression are still unknown. Our study aimed to investigate programmed death-ligand 1 (PD-L1) expression in spontaneously regressed testicular germ cell tumors, exploring the link between the immune response and spontaneous regression. From a sample of 356 testicular germ cell tumors, we singled out 5 completely regressed and 6 partially regressed tumors. In four out of six cases with partial regression, a residual seminoma component was found, while in the remaining two cases, an embryonal carcinoma component was found. Comparisons were made with 20 pure seminomas and 20 mixed germ cell tumors (MGCTs). A semiquantitative immunohistochemical analysis of PD-L1 expression in tumor cells and intra/peritumoral lymphocytes was performed. There was no PD-L1 expression in tumors with complete regression. All partially regressed tumors showed expression in intra/peritumoral lymphocytes within the tumor remnants. Expression was significantly more frequent in pure seminomas compared to MGCTs (*P* ═ 0.004). A positive correlation was demonstrated between the seminoma component and the proportion of PD-L1 positive lymphocytes, with a Kendall Tau-b coefficient of 0.626 (*P* < 0.001). Tumor cells showed PD-L1 expression in three MGCTs within the embryonal carcinoma component. Our results support an immunological mechanism of spontaneous tumor regression, with the strongest potential in testicular tumors containing seminoma components. However, further research is necessary to determine the role of PD-L1 ligand more precisely in the microenvironment of spontaneously regressed tumors.

## Introduction

Testicular germ cell tumors (TGCTs) are the most common malignant neoplasms in men aged 15–40 [1]. In 2020, the age-standardized incidence rate was highest in Europe and Oceania (≥7 per 100,000), while the age-standardized mortality rate was highest in Central and South America (0.8 and 0.5 per 100,000, respectively) [2]. Despite the increasing global incidence in recent decades, it is encouraging that more than 90% of patients with metastases have a curable disease, with an excellent response to chemotherapy based on bleomycin, etoposide, and cisplatin (BEP) [3]. However, 20%–30% of patients relapse after first-line chemotherapy. Of those who relapse, 20%–60% are cured with salvage chemotherapy, which includes paclitaxel, ifosfamide, or vinblastine in addition to cisplatin. Unfortunately, the rest of the patients have platinum-refractory disease [4, 5]. Therefore, understanding resistance to existing cytostatics and seeking new therapeutic strategies are the main focuses of recent studies [6, 7].

Tumor progression is regulated by the interaction between tumor cells and their microenvironment. Inflammatory infiltration is a well-known feature of TGCTs, especially seminomas, and correlates with good clinical outcomes [8, 9]. This points to the possible benefit of enhancing the natural immune response using immune checkpoint inhibitors [10–12]. Programmed death-ligand 1 (PD-L1) positive lymphocytes in TGCTs are considered a positive prognostic factor with good progression-free and overall survival [13]. In contrast, PD-L1 positive tumor cells are associated with poor progression-free and overall survival, as well as with negative prognostic characteristics, such as pT2-3 stage, ≥3 metastatic sites, non-pulmonary visceral metastases, and frequent lymphovascular invasion [11, 12]. However, the results of previous clinical studies and individual case reports show poor efficacy of checkpoint inhibitors in TGCTs [14].

Regressed tumors comprise around 5% of TGCTs [15], and are described as a separate entity in the last two editions of the World Health Organization (WHO) classification of testicular tumors [16, 17]. These are tumors that have regressed spontaneously without any therapy. Regression can be complete or partial [18]. The term “burned-out tumor” is often used as a synonym for completely regressed tumors. Despite the regression of primary testicular tumors, patients often present with metastatic disease, where metastases are the only cause of symptoms and the beginning of the diagnostic work-up [15, 19]. Primary extragonadal tumors are a potential differential diagnosis, but given their rarity, every finding of an extragonadal germ cell tumor is interpreted as a metastasis originating from the testis until proven otherwise [20, 21].

Along with melanomas, lymphomas, leukemias, neuroblastomas, and renal cell carcinomas, TGCTs represent tumors in which spontaneous regression occurs most often, though it has been described in all tumor types [22, 23]. Several theories have been proposed about the mechanisms of spontaneous regression, with the most attention given to an intense immune response and ischemia resulting from hypoperfusion and high metabolic demands of the tumor tissue [23–26]. Historical examples of tumor regression following acquired infection highlight the importance of the inflammatory response in limiting tumor progression [23, 27]. Recent research on humanized mouse models confirms the active role of CD8+ and CD4+ lymphocytes in the spontaneous regression of allogeneic B cell lymphoma [28].

However, the variable response to immunotherapy with checkpoint inhibitors in different types of tumors, especially TGCTs, points to multiple mechanisms of tumor regression, which do not depend exclusively on lymphocytic inflammation. This also suggests the possible multiple roles of immune checkpoints and/or their ligands. The objective response rate (ORR) to immunotherapy with anti-PD-1/L1 inhibitors in patients with advanced tumors of different origins is estimated at only 24% [29]. Therefore, predictive biomarkers are very important for the most accurate stratification of patients who will respond to the therapy. PD-L1 expression is the most frequently investigated biomarker [30, 31], but it has not been investigated in regressed TGCTs. In our research, we examined its expression in tumor cells and tumor-infiltrating lymphocytes (TILs) of spontaneously regressed TGCTs, pure seminomas, and mixed TGCTs (MGCTs) in the context of the immune mechanism of spontaneous tumor regression.

## Materials and methods

### Sample collection

The pathohistological database of the Clinical Department of Pathology and Cytology Ljudevit Jurak was searched with a special algorithm designed by I.P. using the keywords “regressed,” “fibrosis,” “testis,” and “burned-out tumor,” covering the period from January 1^st^ 2011, to January 1^st^ 2021. Out of 356 germ cell neoplasia in situ (GCNIS)-related TGCT diagnoses, there were 11 cases with changes classified as regressed TGCT. Inclusion criteria for the diagnosis included more than 80% fibrous proliferation with sparse lymphocytic infiltration and at least GCNIS area in the peritumoral or regressed/burned-out tumor. Samples of regressed tumors were matched by age with samples from 20 pure seminomas and 20 MGCTs with at least an embryonal carcinoma (EC) and teratoma component in each tumor. All histotypes were determined following the WHO criteria [32]. Samples were reanalyzed microscopically by two uropathologists (B.K. and M.U.), and clinical data, including the age of patient diagnosis and disease stage, were obtained from the Urology Department database. One representative paraffin block was chosen, containing tumor/regressed area and GCNIS area, and prepared for immunohistochemistry.

### Morphometry and protein expression analysis

Samples were cut at 4 µm, deparaffinized, and antigen retrieval was performed (incubation with 10% BSA for 20 min). Sections were incubated with primary antibodies overnight at 4 ^∘^C. The primary antibodies used were Ventana SP263 and SP142 PD-L1 clones. This was followed by incubation with 3% H_2_O_2_ to block endogenous peroxidase, and then incubation with a secondary antibody on the Ventana BenchMark GX platform. The signal was visualized using DAB chromogen. Slides were counterstained with hematoxylin and embedded. Appropriate positive and negative controls were used in staining (normal tonsil tissue). Morphometric analysis for protein expression was performed by two pathologists (B.K. and M.U.) and all disagreements were resolved by a joint committee. Expression of proteins was analyzed in three compartments: each component of tumor tissue, germ cell neoplasia in situ, and regressed areas. The staining signal (brown in color) was noted as cytoplasmic or membranous in tumor cells, as well as in the intra/peritumoral inflammatory cells, i.e., TILs. The staining percentage was scored from 0–3: 0 (negative tumor/inflammatory cells), 1 (>0 to <20% positive tumor/inflammatory cells), 2 (≥20% to <50% tumor/inflammatory cells), and 3 (≥50% tumor/inflammatory cells).

### Ethical statement

Our study has been approved by the Research Ethics Committee of the Clinical Hospital Center Sestre milosrdnice in the context of CERRM project and has adhered to the principles established in the World Medical Association Declaration of Helsinki. We did not use the personal data of the patients and their identity has not been compromised at any time during the research.

### Statistical analysis

The data were statistically analyzed using JASP 0.18.1.0 software (University of Amsterdam, Amsterdam, The Netherlands). The normality of the distribution of the variables was tested using the Shapiro–Wilk test. Differences between categorical variables were examined using the χ^2^ test and Fisher’s exact test when the number of samples was <40. The Kruskal–Wallis test and Dunn’s post hoc test with Tukey’s correction were used to compare ordinal variables, while continuous variables were compared using one-way analysis of variance. Kendall’s Tau-b correlation coefficient was used to examine the relationship between a dichotomous nominal variable and an ordinal variable. All tested samples were independent of each other. Results were considered statistically significant when *P* < 0.05.

## Results

Over the ten-year period of searching our archive, we found 356 TGCTs among which 11 patients had areas of regression. Five patients showed complete regression (“burned-out” tumors), while six patients showed partial regression areas with preserved tumorous tissue, four seminomas, and two ECs) (Table 1). In all 11 cases of regressed tumors, we found GCNIS, in addition to the hyaline scar and mononuclear infiltrate.

**Table 1:** Clinical and immunohistochemical features of regressed tumors

**Patients**	**Histology**	**TNM (stage)**	**Age**	**PD-L1 tumor cells**	**PD-L1 lymphocytes**
1	Burned-out	TN0M1 (III)	19 min	0	0
2	Burned-out	TN0M1 (III)	27	0	0
3	Burned-out	TN0M1 (III)	34	0	0
4	Burned-out	TN0M0 (pT0)	41 max	0	0
5	Burned-out	TN0M0 (pT0)	*	0	0
6	Fibrosis+ seminoma – 20%	T2N1M0 (II)	44 max	0	≥50%
7	Fibrosis+ seminoma – 60%	T1N0M0 (I)	27	0	≥50%
8	Fibrosis+seminoma – 70%	T1N0M0 (I)	32	0	≥50%
9	Fibrosis+seminoma – 30%	T1N0M0 (I)	23 min	0	≥50%
10	Fibrosis+EC--30%	T2N0M1 (III)	30	0	≥10%
11	Fibrosis+EC--70%	T1N0M0 (I)	34	0	≥10%

The lowest (min) and highest (max) ages are marked. *Data not available.

**Table 2:** Clinical and immunohistochemical features of pure seminomas

**Patients**	**TNM (stage)**	**Age**	**PD-L1 tumor cells**	**PD-L1 lymphocytes**
12	T1N0M0 (I)	21	0	≥50%
13	T1N0M0 (I)	34	0	≥50%
14	T2N0M0 (I)	32	0	≥50%
15	T1N1M0 (II)	28	0	≥50%
16	T2N1M0 (II)	31	0	≥50%
17	T3N0M1 (III)	44	0	≥50%
18	T1N0M0 (I)	26	0	≥50%
19	T2N0M0 (I)	39	0	≥50%
20	T1N0M0 (I)	47	0	≥50%
21	T2N0M0 (I)	25	0	≥50%
22	T1N1M0 (II)	34	0	≥50%
23	T3N1M0 (II)	22	0	≥50%
24	T1N0M0 (I)	17 *min*	0	≥50%
25	T1N0M0 (I)	27	0	≥50%
26	T1N0M1 (III)	42	0	≥50%
27	T1N0M0 (I)	51 *max*	0	0
28	T1N0M0 (I)	38	0	≥50%
29	T3N0M0 (I)	43	0	≥50%
30	T1N1M0 (II)	20	0	0
31	T2N0M0 (I)	32	0	≥50%

The lowest (min) and highest (max) ages are marked.

### Clinical data

**Figure 1.**
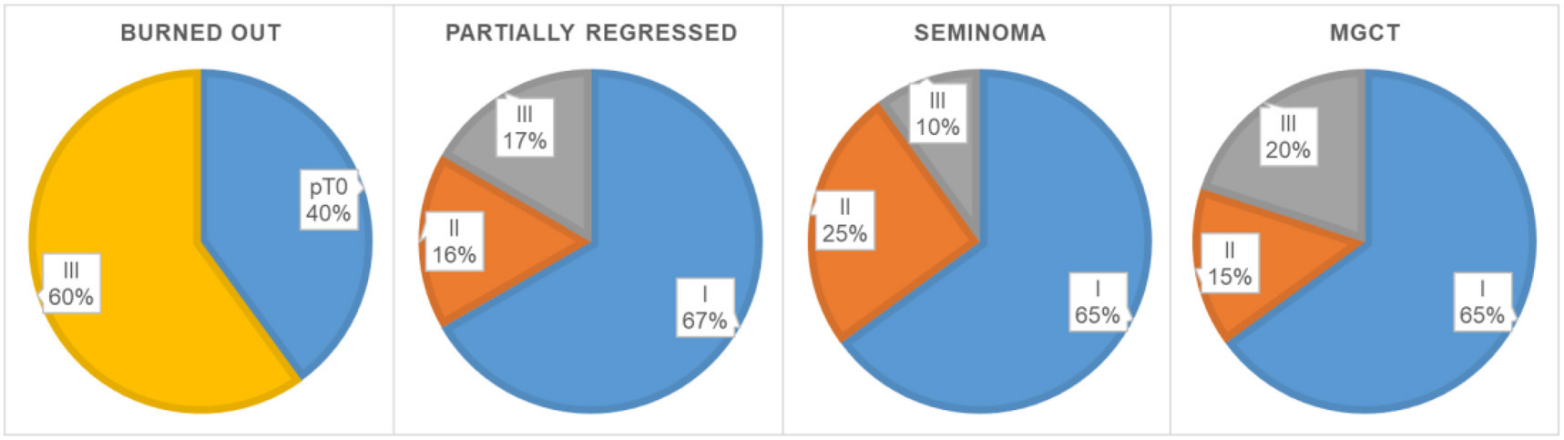
**Frequencies for TNM prognostic groups.** MGCT: Mixed germ cell tumor.

The age distribution of the patients was very uniform (*P* ═ 0.954), with means and standard deviations (SD) in the burned-out, partially regressed, seminoma and MGCT groups as follows: 30.3 years (SD 9.4), 31.7 (SD 7.2), 32.7 (SD 9.6), and 32.5 (SD 6.7), respectively. The marginal age values of each group are marked in Tables 1–3. According to the TNM prognostic groups, patients with burned-out tumors differed significantly compared to the partially regressed (*P* ═ 0.047), seminoma (*P* < 0.001), and MGCT patients (*P* ═ 0.003). They presented either with distal metastases and a testicular scar (stage III) or only with a testicular scar (pT0). There was no statistically significant difference when comparing TNM prognostic groups of partially regressed tumors, seminomas, and MGCTs with each other. Tables 1– 3 and Figure 1 show in detail the frequency of certain stages of the disease among the patients.

**Table 3:** Clinical and immunohistochemical features of mixed germ cell tumors

**Patients**	**Histology**	**TNM (stage)**	**Age**	**PD-L1 tumor cells**	**PD-L1 lymphocytes**
32	MGCT-EC+T	T2N0M0 (I)	27	0	≥20% (EC)
33	MGCT-EC+T+S	T1N0M0 (I)	30	0	≥20% (EC)+ ≥50% (S)
34	MGCT-EC+T+S+YS	T1N0M0 (I)	41	10% (EC)	≥5% (EC)
35	MGCT-EC+T+YS	T1N0M0 (I)	37	0	≥50% (EC)
36	MGCT-EC+T+YS	T3N1M0 (II)	43 *max*	0	≥50% (EC/YS)
37	MGCT-EC+T+YS	T2N0M1 (III)	40	0	0
38	MGCT-EC+T	T1N0M0 (I)	35	0	≥50% (EC)
39	MGCT-EC+T	T2N0M0 (I)	42	15% (EC)	0
40	MGCT-EC+T+S+YS	T2N0M0 (I)	25	0	≥50% (EC/S)
41	MGCT-EC+T+YS	T2N1M1 (III)	31	0	0
42	MGCT-EC+T+YS	T1N1M0 (II)	38	5% (EC)	0
43	MGCT-EC+T+YS	T3N0M0 (I)	39	0	≥20% (EC/YS)
44	MGCT-EC+T+S	T1N0M0 (I)	28	0	≥20% (EC)+ ≥50% (S)
45	MGCT-EC+T	T1N0M0 (I)	34	0	≥50% (EC)
46	MGCT-EC+T+YS	T2N0M1 (III)	27	0	≥50% (EC/YS)
47	MGCT-EC+T+CHO	T3N0M1 (III)	19 *min*	0	0
48	MGCT-EC+T	T1N0M0 (I)	27	0	0
49	MGCT-EC+T+YS	T1N0M0 (I)	33	0	≥50% (EC)
50	MGCT-EC+T+S+YS	T3N1M0 (II)	29	0	10% (EC+YS)
51	MGCT-EC+T+S+YS	T2N0M0 (I)	25	0	0

The lowest (min) and highest (max) ages are marked. MGCT: Mixed germ cell tumor; EC: Embryonal carcinoma; T: Teratoma; S: Seminoma; YS: Yolk sac.

### There was no expression of PD-L1 in burned-out testicular tumors

**Figure 2.**
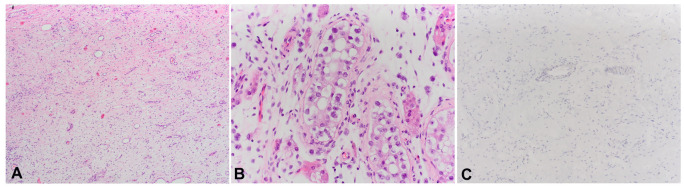
(A) Histologically regressed areas are composed of fibrous tissue with small vessels and scattered lymphocytes (40xHE); (B) Germ cell neoplasm in situ (400xHE); (C) Negative PD-L1 staining (100xPD-L1). PD-L1: Programmed death-ligand 1.

PD-L1 positive lymphocytes were not found in any cases of burned-out tumors (Figure 2), in contrast to partially regressed tumors, seminomas, and MGCTs. In pure seminomas, the expression was statistically significantly more frequent compared to MGCTs, while there was no statistically significant difference when comparing partially regressed tumors with pure seminomas and MGCTs (Tables 4 and 5). In all cases with positive reactions to the PD-L1 antibody, the signal was detected in the cytoplasm of the cells. Notably, in all cases where they had been found, GCNIS cells were PD-L1 negative. In all six partially regressed tumors, PD-L1 positivity was detected on intra/peritumoral lymphocytes, with the reaction being more intense in cases with residual seminoma (Table 1). In the pure seminoma control group, PD-L1 positivity was determined in 18 out of 20 cases, where in each case more than 50% of intra/peritumoral lymphocytes were stained (Table 2). A seminoma component was found in 6/20 MGCTs (Table 3, patients 33, 34, 40, 44, 50, 51). PD-L1 positive lymphocytes in the seminoma component were detected in 3 of those cases (33, 40, 44) with proportions ≥50%. An EC component was found in all 20 MGCTs, but the lymphocytes of the same component were positively stained in 13 cases, with more than 50% of the lymphocytes stained in 7 of these 13 cases. PD-L1 positive lymphocytes in the yolk sac components were found in 4/12 (33,3%) cases of MGCTs that contained yolk sac tumor tissue (patients 36, 43, 46, 50). In only one case (patient 47), a choriocarcinoma component was found, but without a PD-L1 positive reaction. Also, the teratoma component of MGCTs, which was determined in all 20 cases, did not show any type of PD-L1 positivity.

**Table 4:** Dunn’s post hoc comparisons for PD-L1 positive lymphocytes

**Comparison**	***P***
Burned-out – partially regressed	0.002
Burned-out – seminoma	<0.001
Burned-out – MGCT	0.010
Partially regressed – seminoma	0.272
Partially regressed – MGCT	0.120
Seminoma – MGCT	0.004

MGCT: Mixed germ cell tumor; PD-L1: Programmed death-ligand 1.

**Table 5:** Frequencies for PD-L1 positive lymphocytes

**Histology**	**PD-L1 lymphocytes**	**Frequency (%)**
Burned-out (*N* ═ 5)	0	5 (100%)
	>0% –<20%	0
	≥20%–<50%	0
	≥50%	0
Partially regressed (*N* ═ 6)	0	0
	>0%–<20%	2 (33.3%)
	≥20%<50%	0
	≥50%	4 (66.7)
Seminoma (*N* ═ 20)	0	2 (10%)
	>0%–<20%	0
	≥20%–<50%	0
	≥50%	18 (90%)
MGCT (*N* ═ 20)	0	7 (35%)
	>0%–<20%	2 (10%)
	≥20%–<50%	2 (10%)
	≥50%	9 (45%)

MGCT: Mixed germ cell tumor; PD-L1: Programmed death-ligand 1.

### A high proportion of PD-L1 positive lymphocytes was a very common feature of seminoma

**Figure 3.**
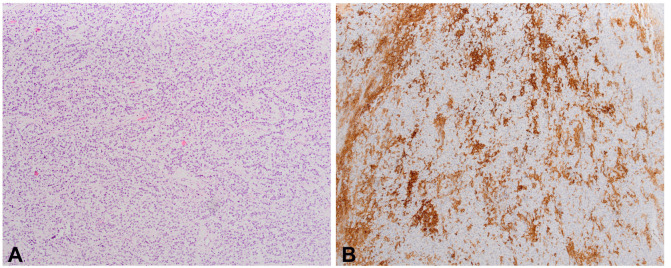
(A) Histologically seminoma is composed of seminoma cells in-between with fibrous septa and dense lymphocytes (100xHE); (B) Positive PD-L1 staining in the lymphocytes (200xPD-L1). PD-L1: Programmed death-ligand 1.

**Figure 4.**
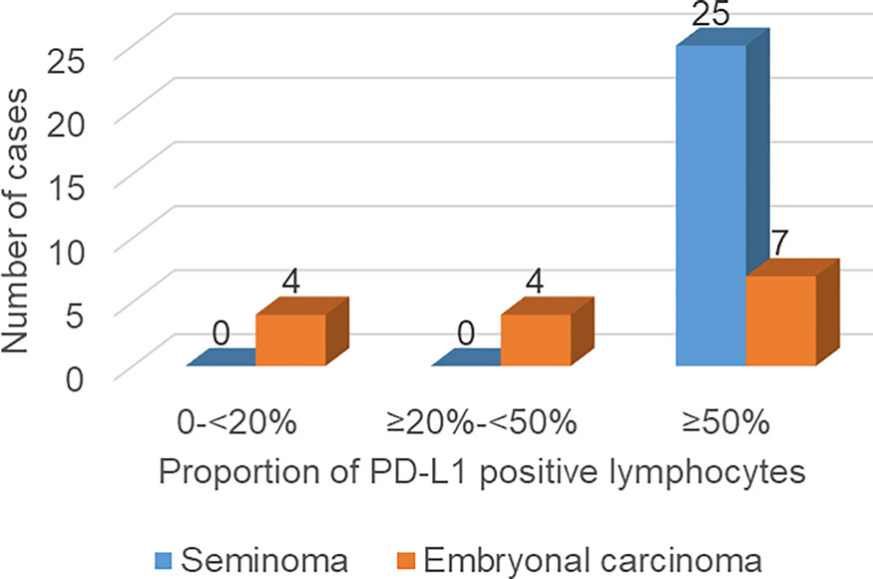
**Frequencies of seminomas and ECs with different proportions of PD-L1 positive lymphocytes.** PD-L1: Programmed death-ligand 1; EC: Embryonal carcinoma.

**Figure 5.**
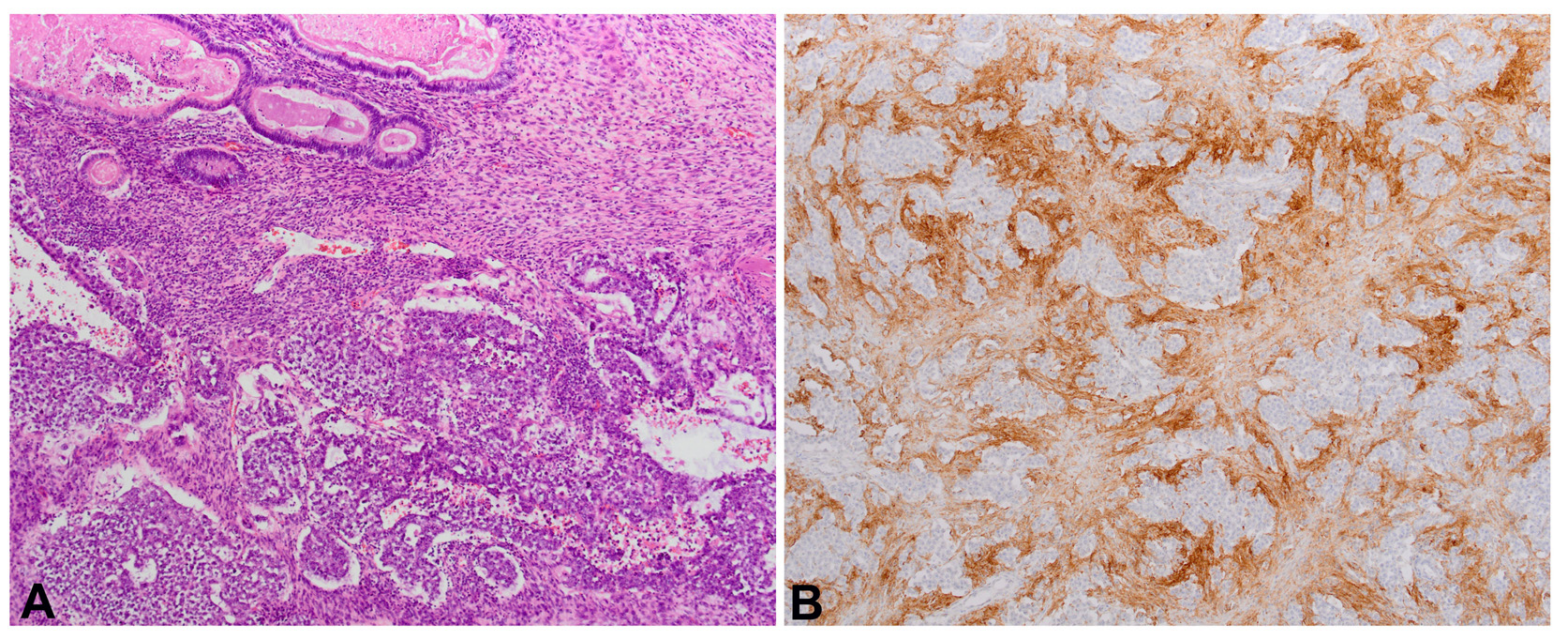
(A) Teratocarcinoma components, composed of mature teratoma elements and atypical cells of EC (100xHE); (B) Positive PD-L1 staining in the lymphocytes (100xPD-L1). PD-L1: Programmed death-ligand 1; EC: Embryonal carcinoma.

**Figure 6.**
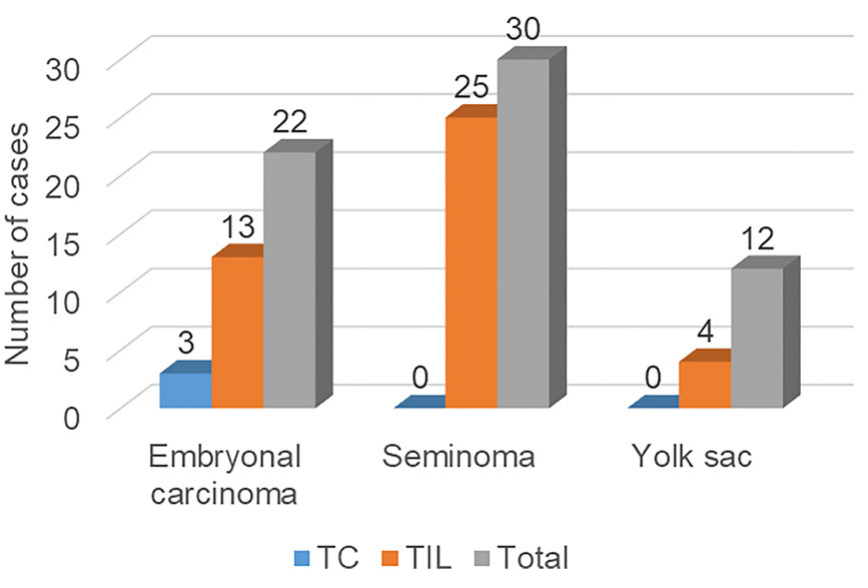
**Frequencies of PD-L1 positive tumor cells (TC) and PD-L1 positive lymphocytes (TIL) among embryonal carcinomas, seminomas, and yolk sac tumors.** PD-L1: Programmed death-ligand 1; TC: Tumor cell; TIL: Tumor-infiltrating lymphocyte.

Among a total of 51 examined cases, EC tissue was found in 22 cases (43%). In 15 of these 22 cases (68%), a positive reaction to the PD-L1 antibody was detected, with tumor cells stained in 2 cases (patients 39 and 42), intra/peritumoral lymphocytes stained in 12 cases, while both tumor cells and lymphocytes positive in one case (patient 34). Seminoma tissue was found in 30/51 cases (59%). In 25 of these 30 cases (83%), the reaction to the PD-L1 antibody was positive, with only intra/peritumoral lymphocytes stained (Figure 3), always in a proportion of ≥50%. The proportion of PD-L1 positive lymphocytes in ECs was significantly lower (*P* < 0.001) compared to seminomas (Figures 4 and 5). Kendall’s Tau-b correlation coefficient between the seminoma component and the proportion of PD-L1 positive lymphocytes was 0.626 with *P* < 0.001. On the other hand, there was no statistically significant difference when comparing the frequency of EC and seminoma that show a positive reaction to the PD-L1 antibody among a total number of EC (*N* ═ 22) and seminoma (*N* ═ 30) cases, respectively (*P* ═ 0.2 for PD-L1 positivity in lymphocytes, *P* ═ 0.085 for PD-L1 positivity in tumor cells). The same comparison of frequency between the yolk sac component (*N* ═ 12), with the EC and seminoma shows that PD-L1 positive lymphocytes are a significantly more frequent feature of seminoma (*P* ═ 0.02), while between yolk sac tumor and EC, there is no significant difference in the PD-L1 positivity on either lymphocytes (*P* ═ 0.075) or tumor cells (*P* ═ 0.537) (Figure 6).

## Discussion

Although spontaneous tumor regression is a rare phenomenon with an estimated frequency of one case in every 60,000 to 100,000 tumor cases, it has been described in almost all types of solid and hematological neoplasms [22, 25]. A comparative analysis of the results from a systematic and a historical review [15, 19] shows that by 2020, at least 78 articles had been published with describing 184 cases of spontaneous regression of TGCTs. Choriocarcinoma was previously considered the most common subtype showing regression, but cases published so far indicate that regression most frequently occurs in seminomas [15, 19]. This is supported by our results with four cases with residual seminoma and two with EC.

The finding of a scar in the testicle requires careful interpretation, given that, in addition to tumor regression, there are other causes of its formation, such as trauma or vascular diseases [33]. In all 11 of our cases, we found GCNIS in the surrounding tissue, which is considered one of the most specific diagnostic criteria of regression [18, 34]. Histological interpretation of partial regression is also challenging, as residual tumor tissue does not always have to be consistent with the regressed tumor, and metastases are not always histologically consistent with the primary tumor [18]. Unlike the cohorts of other authors in which cases with complete regression prevailed, our study confirmed six cases of partial and five cases of complete regression. It is considered that in more than 90% of regressed tumors, the primary tumors are completely regressed with distal metastases [19], which we observed in just three cases (patients 1–3). On the other hand, in only one case with partial regression (patient 10), there was a metastatic disease, with residual EC in the testis.

Lymphocytic infiltration is a well-known feature of TGCTs, with a greater extent in seminomas compared to non-seminomas [8]. It correlates with a good clinical outcome and a lower risk of relapse for seminomas [9]. Balzer and Ulbright [18] found a variable lymphoplasmacytic infiltrate in 37 out of 42 (88.1%) samples of regressed TGCTs, concluding that the immune reaction could play a role in tumor regression. This theory is supported by many reported cases of tumor regression following acquired infections and febrile conditions, assuming it occurred due to a strong immune response triggered by the infection [23, 24]. Programmed cell death protein-1 (PD-1, also known as CD279) is an immune checkpoint membrane receptor that is one of the markers of over-activated, i.e. exhausted, immune cells, including T and B lymphocytes, macrophages, dendritic cells, and monocytes [35, 36]. Interaction with its ligand PD-L1 (also known as CD274), sends inhibitory signals to the nucleus, resulting in the blockade of receptor-mediated cytotoxicity and reduction of cellular proliferation, thus limiting the immune response [37]. PD-L1 transmembrane glycoprotein is expressed on tumor-associated stromal cells, such as macrophages, T and B lymphocytes, and dendritic cells, but also on tumor cells, which is considered an adaptive tumor mechanism to avoid the immune response. After the first anti-PD-1 antibody, pembrolizumab, was approved for the treatment of metastatic or unresectable melanoma in 2014, other PD-1 and PD-L1 inhibitors soon followed [36].

In a recent systematic review of the efficacy of anti-PD-1/PD-L1 monotherapy across 31 tumor types, Mao et al. [38] reported the highest ORR in mismatch repair-deficient colorectal cancer (ORR 38.8%) and mucosal melanoma (ORR 37.0%), while in germ cell tumors the ORR was 0%. So far, two phase II clinical trials on the effectiveness of anti-PD-1/PD-L1 inhibitors in patients with advanced germ cell tumors have been completed [39, 40], two phase II trials were terminated [41, 42], while there are one phase I [43] and five phase II [44–48] ongoing clinical trials.

PD-L1 expression is one of the most frequently investigated predictive biomarkers for therapy with anti-PD-1/PD-L1 inhibitors [49]. According to the results of recently published meta-analyses, the sensitivity and specificity of immunohistochemical detection of PD-L1 expression on tumors and/or immune cells as biomarkers for responders to anti-PD-1/L1 immunotherapy are both about 60% [30, 50]. However, none of the included studies in these meta-analyses examined the value of PD-L1 expression as a biomarker in TGCTs.

In our research, we found PD-L1 expression in 39 out of a total of 51 analyzed samples, i.e., in 76.47% of cases. Observing each histological component separately, seminomas, ECs, and yolk sac tumors expressed PD-L1 in 83.3%, 68.2%, and 33.3% of cases, respectively, while expression was not detected in the choriocarcinoma and teratoma components. In two ECs, expression was found in tumor cells, in one case, expression was found in both tumor cells and lymphocytes, while in the remaining cases, expression was seen only in lymphocytes. In seminomas and yolk sac tumors, only lymphocytes were positively stained.

Not precisely defining the type of cells, Fankhauser et al. [10] found PD-L1 expression in 73% of seminomas and 64% of non-seminomas among a total of 479 samples. In our cohort, positivity was observed in 83.3% of seminomas and in 34.6% of non-seminomas. Previous studies showed that choriocarcinomas most often express PD-L1 on tumor cells [11, 12, 51]. Our results point to the rare occurrence of PD-L1 expression on tumor cells, with membranous/cytoplasmic staining detected on EC cells in three cases, which is in concordance with the previous studies [11, 12] where EC is the second most common histological type with PD-L1 expression on tumor cells. We note, however, that in our cohort, there was only one sample with choriocarcinoma component, preventing us from concluding the phenotype of choriocarcinoma.

Similar to our results, Chovanec et al. [13] found the highest frequency and proportion of PD-L1 lymphocytes in seminomas (95.9% and 61.0%, respectively) and ECs (91.0% and 42.4%, respectively). However, they also found PD-L1 lymphocytes in choriocarcinoma and teratoma samples. Our results show the highest frequency of PD-L1 positive lymphocytes in seminomas, with a decreasing frequency in ECs (*P* ═ 0.2) and yolk sac tumors (*P* ═ 0.02). Regarding the proportion of PD-L1 positive lymphocytes, we observed a significantly stronger reaction in seminomas compared to ECs (*P* < 0.001) and we found a strong correlation between the seminoma component and the proportion of PD-L1 positive lymphocytes (Kendall’s Tau-b correlation coefficient ═ 0.626, *P* < 0.001).

The difficulty in determining the exact percentage of PD-L1 positive lymphocytes in yolk sac components of the MGCTs limited our ability to compare the proportion of PD-L1 lymphocytes in yolk sac tumors with seminomas and ECs. Zhang et al. [52] detected PD-L1 positive tumor cells in four testicular yolk sac tumors, while in two of those, they found PD-L1 positive lymphocytes, in contrast to our results.

Interestingly, we did not detect PD-L1 expression in any tumor with complete regression, while the expression on lymphocytes was confirmed in all six cases with partial regression. This can be interpreted in several ways. Assuming that regression is a dynamic and directed immune process with complete regression as the final outcome, PD-L1 positive lymphocytes could be considered an integral part of the executive mechanism by which regression progresses. Therefore, the presence of PD-L1 positive lymphocytes is expected in partially regressed tumors where the regression process is still ongoing, in contrast to completely regressed tumors where the regression has already ended. With regard to the turnover of cells in the scar in which tumor antigens are no longer present, we cannot rule out that these are new clones of recruited lymphocytes with a different phenotype compared to the anti-tumor clones that have disappeared. On the other hand, considering the absence of PD-L1 positive lymphocytes in completely regressed tumors, their presence in tumors that have only partially regressed can be identified as a brake signal that prevents the regression process from ending with complete regression as the final result. For now, we can only speculate about these explanations.

The poor antitumor efficacy of PD-1/PD-L1 inhibitors may indicate the multiple yet undiscovered roles of these molecules. For example, it is known that PD-L1 also plays a role in the survival of CD8+ lymphocytes during the immune response [9]. Furthermore, better efficacy of anti-PD-1/PD-L1 inhibitors has been observed in tumors with a high tumor mutational burden (TMB) [30, 38, 50], which is an additional explanation for their poor efficacy in TGCTs, known to have low TMB [9]. Despite the overall better therapeutic response in tumors that express PD-L1 either on tumor or immune cells [29, 53], the efficacy of anti-PD-1/PD-L1 inhibitors is not at a satisfactory level, especially in TGCTs. This indicates the need to find other therapeutic targets to overcome chemotherapy and immunotherapy resistance.

In addition to the small number of cases, our research has several shortcomings. First, we did not have data on the localization of metastases. Moreover, the patients were not clinically followed, so we could not analyze the survival and clinical outcome of regressed tumors. Additionally, given the absence of a more detailed immunophenotypic characterization of the tumor microenvironment, we cannot conclude the precise role of the PD-L1 ligand in the regression process. Finally, for immunohistochemical staining, we used two different antibodies, of which the SP142 has a lower sensitivity than the SP263 [54].

## Conclusion

Our results suggest that seminoma is the most common subtype of TGCT with spontaneous regression. We demonstrated the absence of PD-L1 expression in completely regressed TGCTs. The presence of PD-L1 positive lymphocytes in tumors with partial regression supports the immunological theory of spontaneous regression, although the design of our study does not allow us to draw more precise conclusions about the role of the PD-L1 ligand in the mechanism of spontaneous tumor regression. We also confirmed the highest frequency of PD-L1 positive lymphocytes in seminomas and proved the existence of a positive correlation between the proportion of PD-L1 positive lymphocytes and seminomas.
